# Role of Optical Phonons and Anharmonicity in the Appearance of the Heat Capacity Boson Peak-like Anomaly in Fully Ordered Molecular Crystals

**DOI:** 10.1021/acs.jpclett.2c01224

**Published:** 2022-06-02

**Authors:** Alexander I. Krivchikov, Andrezj Jeżowski, Daria Szewczyk, Oxsana A. Korolyuk, Olesya O. Romantsova, Lubov M. Buravtseva, Claudio Cazorla, Josep Ll. Tamarit

**Affiliations:** † Verkin Institute for Low Temperature Physics and Engineering of the National Academy of Sciences of Ukraine, 47 Nauky Avenue, Kharkiv 61103, Ukraine; ‡ Institute of Low Temperature and Structure Research, Polish Academy of Sciences, 2 Okólna Strasse, 50-422 Wrocław, Poland; § Grup de Caracterizació de Materials, Departament de Fisica, EEBE, and Barcelona Research Center in Multiscale Science and Engineering, Universitat Politècnica de Catalunya, Av. Eduard Maristany, 10-14, 08019 Barcelona, Catalonia, Spain

## Abstract

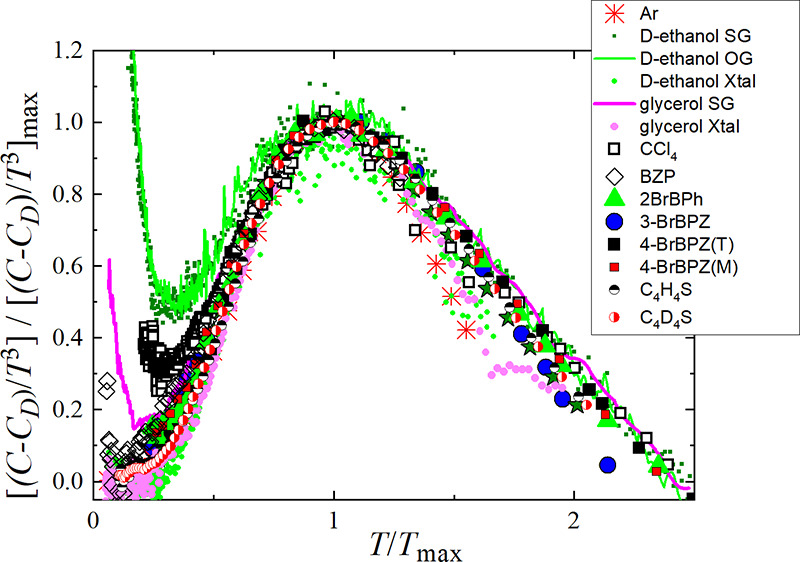

We demonstrate that the heat capacity Boson peak (BP)-like anomaly appearing in fully ordered anharmonic molecular crystals emerges as a result of the strong interactions between propagating (acoustic) and low-energy quasi-localized (optical) phonons. In particular, we experimentally determine the low-temperature (<30 K) specific heat of the molecular crystal benzophenone and those of several of its fully ordered bromine derivatives. Subsequently, by means of theoretical first-principles methods based on density functional theory, we estimate the corresponding phonon dispersions and vibrational density of states. Our results reveal two possible mechanisms for the emergence of the BP-like anomaly: (i) acoustic–optic phonon avoided crossing, which gives rise to a pseudo-van Hove singularity in the acoustic phonon branches, and (ii) piling up of low-frequency optical phonons, which are quasi degenerate with longitudinal acoustic modes and lead to a surge in the vibrational density of states at low energies.

Glasses exhibit a characteristic anomaly in the low-frequency region (≈1 THz) of the vibrational density of states [*g*(ω), VDOS] known as the Boson peak (BP),^1−4^ an excess of vibrational states as determined by the Debye squared frequency law for crystals, which manifests as a peak in the reduced VDOS of the glass [i.e., the *g*(ω)/ω^2^ vs ω representation, where ω is the energy of the vibrational excitation]. The same anomaly appears as a low-temperature (5–20 K) peak in the reduced heat capacity (*C*_*p*_), *C*_*p*_/*T*^3^ versus *T*, in contrast to the constant of the Debye model (*C*_D_ = 12π^4^*R*/5Θ_D_^3^, where Θ_D_ is the Debye temperature of the solid). At even lower temperatures (below ≈1–2 K), the *C*_*p*_ of glasses exhibits a linear dependence on *T*, traditionally explained in terms of quantum tunneling between different system configurations with very close energies [i.e., “two-level” systems (TLS)].^4−6^ Additional glassy anomalies appear also in the thermal conductivity, κ(*T*). At low temperatures, κ first increases with *T*^2^ (rather than with *T*^3^) and subsequently saturates on a plateau that is orders of magnitude lower than the typical κ values found in crystals.^1−3^

Despite the enormous research efforts devoted to the understanding of the glassy state, there is still no consensus about the physical origins of its thermal anomalies. Several theories have been put forward to rationalize the observed phenomenology based on the interactions between soft (localized) and acoustic modes,^7^ heterogeneous elasticity,^8^ local breaking of inversion symmetry,^9^ an equivalence between the BP and van Hove singularity in the crystalline counterparts,^10^ phase transitions in the space of stationary energy points,^11^ transverse vibrational modes associated with defective soft structures in the disordered state,^12^ and random matrix models,^13^ to mention just a few. In all of these theoretical models, disorder always plays a central role.

However, during the past decade several experimental and molecular dynamics studies have also evidenced the existence of glass-like *C*_*p*_ anomalies in perfectly ordered and minimally disordered molecular crystals.^14−28^ These findings suggest that the physical causes of the described anomalies should be more general than previously thought and not exclusive of glasses. It is therefore reasonable to think that by improving our physical understanding of molecular crystals exhibiting minimal or null disorder, which can be carefully and thoroughly analyzed with well-established experimental and computational techniques, we can clarify the apparently “universal” character of the BP and progress in our unsatisfactory comprehension of glasses. In this direction, here we determine the low-temperature heat capacity (0.39 K ≤ *T* ≤ 30 K) of single crystals of benzophenone (C_13_H_10_O, BZP) (*C*_2_ symmetric molecule in the inset of Figure 1) and the stable and metastable crystalline phases of several bromine derivative isomers: 2-, 3-, and 4-bromobenophenone (2-BrBZP, 3-BrBZP, and 4-BrBZP, respectively). These bromine-benzophenones (C_13_H_9_OBr) are isomers that differ in the position (2, 3, and 4, respectively) of the Br atom in one of the phenyl rings (*o*-, *m*- and *p*-bromobenzophenone, respectively). The polymorphism of these materials has been extensively studied by using different experimental techniques^29−42^ (see the Supporting Information for details). For the particular case of 4-BrBZP, we have analyzed the stable monoclinic crystalline phase [4-BrBZP(M)] and a metastable triclinic polymorph [4-BrBZP(T)] that can be supercooled to the lowest temperature considered here. Experimental details of the single-crystal growth and the structural and thermodynamic characterizations are provided in the Supporting Information.

**Figure 1:**
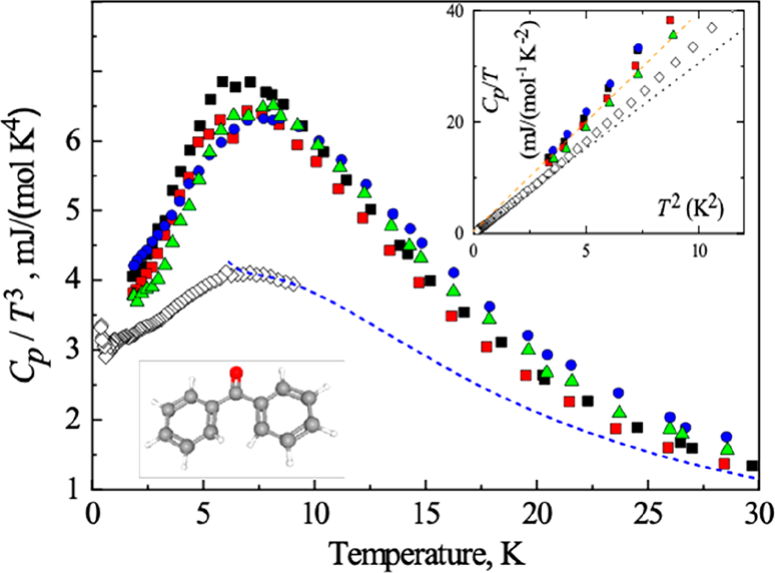
Experimental heat capacity of benzophenone and its bromine derivatives in the reduced representation *C*_*p*_/*T*^3^ vs *T*: black squares for triclinic and metastable 4-BrBZP(T), red squares for monoclinic and stable 4-BrBZP(M), blue circles for 3-BrBZP, green triangles for 2-BrBZP, and empty diamonds for BZP. BZP literature data are shown as blue dotted curves.^32,33^ The bottom left inset shows the BZP molecule. The top right inset shows a plot of *C*_*p*_/*T* vs *T*^2^ within the low-temperature range.

Figure 1 shows our experimental heat capacity results represented in the reduced form *C*_*p*_/*T*^3^ and expressed as a function of temperature. The data clearly evidence a heat capacity BP-like anomaly in all cases, regardless of the symmetry and stable or metastable character of the (fully ordered) crystalline phase. The BP-like maximum occurs at very similar temperatures in all the molecular crystals, *T*_max_, whereas the maximum reduced heat capacity [(*C*_*p*_/*T*^3^)_max_] is approximately 1.6 times lower in BZP than in the brominated compounds. The heat capacity data were fitted to the well-known low-temperature polynomial expansion:
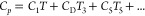
1where *C*_1_ stands for the linear contribution stemming from possible TLS tunneling effects, *C*_D_ the Debye contribution from linear acoustic modes, and *C*_5_ the contribution from other low-energy (soft) modes.^43,44^ The parameters fitted to our experimental data are listed in Table 1.

**Table 1:** Heat Capacity Parameters of Benzophenone (BZP), 2-Bromobenzophenone (2-BrBZP), 3-Bromobenzophenone (3-BrBZP), and 4-Bromobenzophenone (4-BrBZP)^a^

sample	symmetry (stability)	*T*_max_ (K)	*C*_*p*_/*T*^3^(*T*_max_) (J mol^–1^ K^–4^)	*C*_3_ (mJ mol^–1^ K^–4^)	*C*_5_ (mJ mol^–1^ K^–6^)	Θ_D_ (K)
BZP	*P*2_1_2_1_2_1_, *Z* = 4 (s)	7.1 ± 0.2	4.1	3.06 ± 0.05	0.036 ± 0.001	85
2-BrBZP	*P*2_1_/*a*, *Z* = 4 (s)	7.6 ± 0.4	6.46	3.3 ± 0.03	0.09 ± 0.001	83
	*P*2_1_/*c*, *Z* = 4 (m)	7.2 [28]	5.11 [28]	2.7 [28]	0.068 [28]	89
3-BrBZP	*Pbca*, *Z* = 8 (s)	7.6	6.32	4.0 ± 0.03	0.07 ± 0.001	78
4-BrBZP	*P*2_1_/*c*, *Z* = 4 (s)	6.9 ± 0.3	6.42	3.4 ± 0.03	0.11 ± 0.001	82
	*P*1305, *Z* = 2 (m)	6.7 ± 0.3	6.84	3.7 ± 0.05	0.11 ± 0.001	80

a Space group symmetry, number of molecules per unit cell (*Z*), and stable (s) or metastable (m) character of the crystalline phases. *T*_max_ is the maximum of the *C_p_*/*T*^3^ function. Coefficients *C*_3_ and *C*_5_ are from eq 1. Θ_D_ is the Debye temperature deduced from the equation Θ_D_^3^ = 12π^4^*R*/(5*C*_3_).

To unequivocally identify the origin of the BP-like *C*_*p*_ features in BZP-based molecular crystals, we thoroughly analyzed the corresponding vibrational phonon spectra. Experimental determination of phonon dispersions of molecular crystals, ω(*k*), is extremely challenging in practice due to the technical limitations encountered in the growth of single crystals and the small scattering cross section of the involved atomic species. Thus, in this work, we employed theoretical first-principles calculations based on density functional theory (DFT) to estimate the relevant ω(*k*) and *g*(ω) values. In particular, we evaluated the vibrational phonon properties of the parent BZP crystal and the fully ordered stable (monoclinic, M) and metastable (triclinic, T) 4-BrBZP phases. Excellent qualitative agreement between our experiments and DFT calculations was obtained for the heat capacity and Debye temperature of the analyzed molecular crystals (see the Supporting Information and Figure S1).

Density functional theory (DFT) calculations^45^ based on the PBE functional^46^ were performed with VASP software.^47^ Long-range dispersion interactions were captured with the DFT-D3 method.^48^ Wave functions were represented in a plane-wave basis truncated at 650 eV, and a k-point grid of 2 × 2 × 4 (2 × 4 × 4) was employed for integrations within the Brillouin zone (BZ) of the stable BZP and 4-BrBZP phases (metastable 4-BrBZP phase). Phonon calculations were performed within the harmonic approximation by means of density functional perturbation theory calculations (Γ point)^46^ and the small displacement method (full phonon spectrum).^49^ Additional details of our first-principles calculations can be found in the Supporting Information.

Figure 2 shows the results of DFT frozen-phonon calculations^50^ performed for the first optical Γ phonon mode of BZP and 4-BrBZP(T) estimated at normal pressure, which have low energies of 3.44 and 4.31 meV, respectively, and are greatly dominated by Br displacements in the case of 4-BrBZP(T) (i.e., account for ∼50% of the phonon eigenmode). Our first-principles calculations demonstrate the marked anharmonic character of BZP-based molecular crystals. A fourth-order polynomial is necessary to accurately fit the energy curve associated with the static lattice distortion of the phonon eigenmode, which is very shallow and asymmetrical around the origin (i.e., exhibits a nonparabolic phonon potential).^50^ The frozen-phonon potential energy curve estimated for BZP is broader and more asymmetrical than for 4-BrBZP(T), which suggests a higher degree of anharmonicity in BZP. Moreover, the harmonic full phonon spectra calculated at normal pressure for 4-BrBZP(M), 4-BrBZP(T), and BZP all display several imaginary phonon frequency modes, which is also a clear signature of their strong anharmonic character.^50^ To eliminate such vibrational phonon instabilities and to correctly estimate the thermodynamic properties (e.g., heat capacity) within the harmonic approximation, we pressurized BZP and 4-BrBZP (up to ≈2 GPa) in our calculations. The only expected changes deriving from such a pressure-induced stabilization are a generalized increase in the vibrational energy levels [e.g., the first optical Γ phonon mode of 4-BrBZP(T) moves from 4.31 meV at normal pressure to 6.75 meV at ≈2 GPa] and an upward shift in the characteristic temperatures Θ_D_ and *T*_max_.

**Figure 2:**
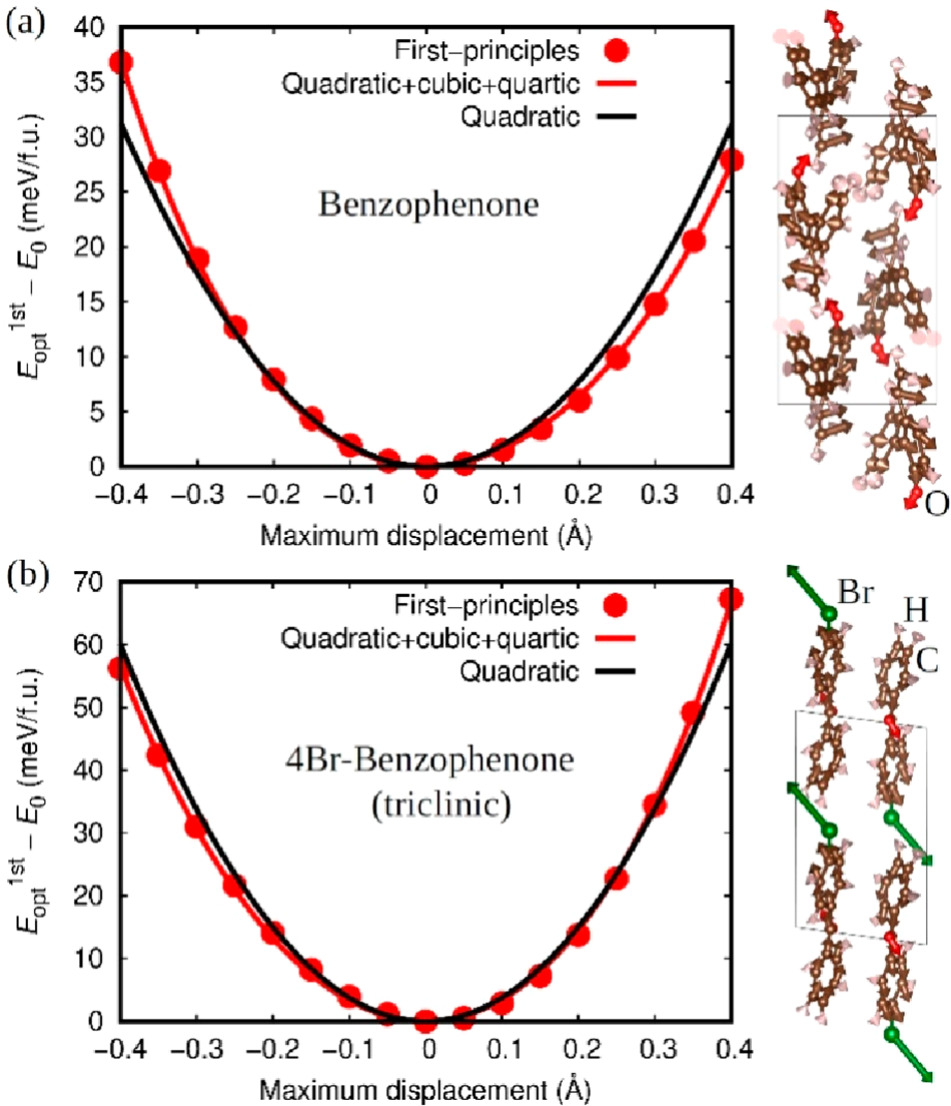
DFT-calculated frozen-phonon potentials for the first optical Γ mode of (a) benzophenone and (b) 4-bromobenzophenone (T). Solid points represent the actual DFT calculations, and solid lines polynomial fits. The atomic displacements involved in the phonon eigenvectors are represented in the margins by solid arrows that are proportional to them.

From the calculated phonon dispersion relations, ω(*k*) (Figure 3b,e,h), we estimated the VDOS [*g*(ω)] and reduced VDOS [*g*(ω)/ω^2^] shown in panels a, d, and g of Figure 3. For each crystal, the energy region relevant to the BP-like anomaly is identified from the first low-energy peak in the reduced VDOS representation. Such key energy regions are highlighted in gold in Figure 3 and occur around 5.9, 5.9, and 4.4 meV in 4-BrBZP(M), 4-BrBZP(T), and BZP, respectively. Meanwhile, panels c, f, and i of Figure 3 show the partial contributions to the VDOS, from which one can clearly appreciate that in 4-BrBZP(M) and 4-BrBZP(T) the Br ions play a dominant role in the frequency region that is relevant to the BP-like anomaly. Thus, different physical mechanisms leading to the appearance of the BP-like anomaly in principle could be expected in 4-BrBZP and BZP.

**Figure 3:**
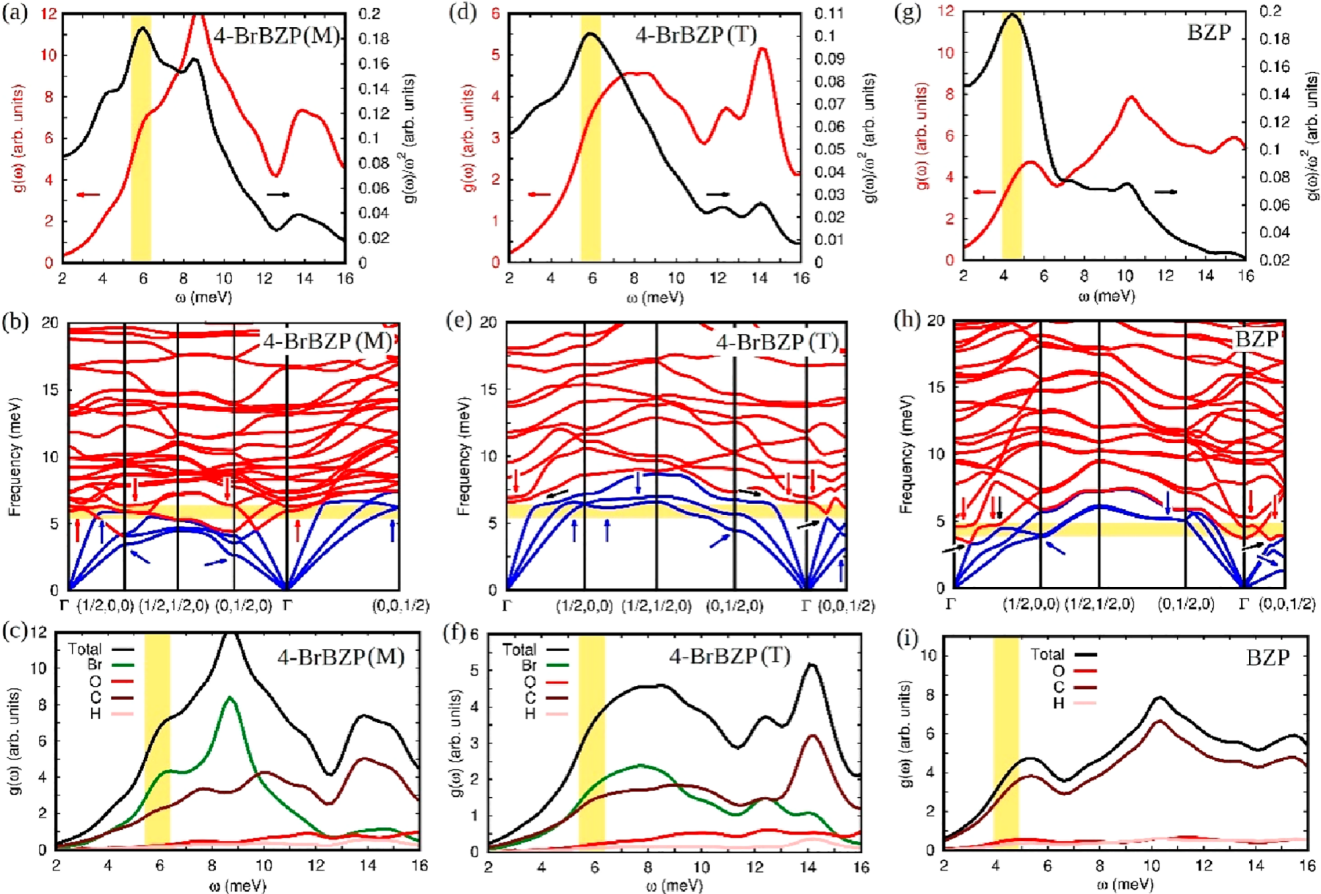
Vibrational phonon properties of 4-BrBZP(M) (stable phase, left panels), 4-BrBZP(T) (metastable phase, central panels), and BZP (right panels) calculated with first-principles methods. (a, d, and g) Vibrational densities of states *g*(ω) in the normal (left *y*-axis) and reduced representations, *g*(ω)/ω^2^ (right *y*-axis). (b, e, and h) Low-energy phonon dispersion relations. (c, f, and i) Partial atomic contributions to *g*(ω). Energy regions highlighted in gold denote those that are relevant to the *g*(ω)/ω^2^ maximum and the BP-like anomaly. Red arrows in panels b, e, and h indicate reciprocal-space regions where optical modes contribute to the BP-like anomaly (quasi-localized optical modes, dω_opt_/d*k* ≈ 0). Black arrows indicate reciprocal-space regions where avoided phonon crossings appear. Blue arrows indicate van Hove singularities in the acoustic branches.

The strict definition of the first van Hove singularity^51^ in the transverse acoustic (TA) branches of a three-dimensional crystal implies the existence of a null frequency dispersion gradient (dω_TA_/d*k* = 0) and an accompanying discontinuity in the first VDOS derivative with respect to energy. [Longitudinal acoustic (LA) branches possess energies that are higher than those of TA branches and are typically neglected.] In real phonon dispersions, however, acoustic branches generally present regions around saddle points and local maxima in which the phonon group velocities are practically null (*v*_TA_ = dω_TA_/d*k* ≈ 0) but do not entail infinite *g*(ω) singularities.^10^ Therefore, here we consider that the *v*_TA_ ≈ 0 regions can be identified as TA van Hove singularities (blue arrows in Figure 3b,e,h). Upon doing so, we find the first van Hove TA singularity in 4-BrBZP(M), 4-BrBZP(T), and BZP appears at energies of 2.6, 3.1, and 1.3 meV, respectively (i.e., lowest-energy blue arrows in Figure 3b,e,h), always well below the characteristic energies of the corresponding BP-like anomalies [i.e., 5.9, 5.9, and 4.4 meV, respectively (Figure 3a,d,g)]. Thus, our theoretical analysis demonstrates that in fully ordered highly anharmonic molecular crystals there is not a direct correlation between the first TA van Hove singularity and the BP-like anomaly.

Panels b, e, and h of Figure 3 show the presence of quasi-localized optical modes in all investigated crystals with energies close to those of the acoustic phonon branches at reciprocal-space points near the first BZ boundaries. Such quasi-localized optical modes are most evident around the high-symmetry point Γ, and their energies are quite close to the BP-like anomaly energy interval (red arrows in Figure 3b,e,h). As we show next, the existence of such low-energy optical phonons fundamentally contributes to the BP-like anomaly in two different ways: directly, through straightforward piling up of low-energy optical modes, and indirectly, through induction of the flattening of acoustic phonon bands.

With regard to 4-BrBZP(T) and BZP (Figure 3e,h) our results unambiguously reveal the phenomenon of avoided crossing between low-energy optical and LA phonon bands^52−57^ (black arrows). This mechanism involves the presence of strong interactions between optical and acoustic modes^52,58−61^ and is most clearly appreciated in 4-BrBZP(T), where the two lowest-energy optical bands (Figure 3e) experience a sudden group velocity increase at approximately one-third of the Γ → (^1^/_2_, 0, 0) reciprocal-space path that is accompanied by a sound flattening of the LA and TA bands over an ample BZ region. Such a flattening gives rise to a pseudo-van Hove singularity that contributes the most to the BP-like anomaly. It is important to emphasize, however, that such a pseudo-van Hove singularity is different from the “classical” TA van Hove singularity^51^ proposed as the origin of the BP anomaly in glasses.^10^ In particular, for the pseudo-van Hove singularity to appear in 4-BrBZP(T), a strong interaction between the acoustic and lowest-energy optical phonon bands is necessary;^62−64^ hence, even if indirectly, the role of optical phonons for the appearance of the BP-like anomaly is critical.

With regard to the stable phase 4-BrBZP(M) (Figure 3b), a “simple” piling up of low-energy optical and LA phonon modes affords an increase in the VDOS that ultimately leads to the appearance of the BP-like anomaly (in this regard, the TA phonon bands are very much irrelevant). In this case, the optical phonon contribution to the BP-like anomaly is dominant (red arrows in Figure 3b). Interestingly, in 4-BrBZP(M), no thorough flattening of the LA and TA bands is observed in contrast to what we found in 4-BrBZP(T). Such an intriguing difference between the two polymorphs originates in the absence of avoided crossing in the stable 4-BrBZP(M) phase, which is a synonym for the absence of strong optic–acoustic phonon interactions. Consequently, some of the lowest-energy optical and LA phonon bands in 4-BrBZP(M) become quasi degenerate. BZP shares traits with the two 4-BrBZP polymorphs because it exhibits both the avoided crossing phenomenon and the accumulation of low-frequency optical modes (Figure 3h).

Our findings about the origin of the BP-like anomaly are in accordance with previous experimental works in which it was proposed that the excess of VDOS was due to the hybridization of both LA and TA branches with localized optical modes,^65,66^ although in the case of highly anharmonic but fully ordered molecular crystals presented here, there is no need to resort to the existence of nanometric inhomogeneous elastic networks or any other type of disorder.^67^ Similarly, a recent work on a strain glass has also dismissed the smearing of the first van Hove TA singularity and the presence of structural disorder as the fundamental origin of the BP-like anomaly.^68^ Likewise, Rufflé et al.^69^ already proposed that the BP anomaly in glasses could be explained by the existence of “non-acoustic vibrational modes” in the terahertz frequency range, in agreement with our rational picture of the BP-like signature.

Our results are consistent with a recent theory based on an anharmonic crystal model in which, regardless of the degree of disorder of the system,^70,71^ the role played by the low-energy optical modes (with energies close to those of the acoustic bands at points near the BZ boundaries) is central. Such a model also predicts that the linear behavior of the specific heat appearing at temperatures below *T*_max_ results from a strong damping of the optical phonons caused by the crystal anharmonicity, an argument that cannot be put to test by our first-principles calculations due to the numerical limitations encountered in the *T* → 0 limit. Nevertheless, our outcomes suggest that an increase in anharmonicity is not necessarily accompanied by an increase in the height of the *C*_*p*_/*T*^3^ BP-like anomaly. Despite the fact that the degree of anharmonicity in BPZ is higher than in 4-BrBZP(T) (Figure 2), the intensity of the BP-like peak is significantly smaller in BZP (Figure 1). We tentatively ascribe the height of the BP-like anomaly in *C*_*p*_/*T*^3^ to the number of vibrational states at the corresponding relevant frequencies. For instance, panels a, d, and g of Figure 3 show very similar VDOS values around the *g*(ω)/ω^2^ maximum for 4-BrBZP(M) and 4-BrBZP(T) (mind the necessary scaling factor of 2 in the latter case for meaningful comparisons) but much smaller values for BZP.

The anharmonic lattice dynamics of BZP and the two 4-Br-BZP polymorphs can also rationalize the behavior of other thermal properties like κ(*T*).^72^ Literature data show ultralow thermal conductivities for Br-BZP and BZP (displaying bell-shaped temperature dependence), which can be qualitatively understood from the low-frequency region of their calculated dispersion relations (Figure 3b,e,h). The interactions between acoustic branches and low-energy optical phonons (the heat carriers in solids), giving rise to the avoided crossing phenomenon, strongly enhance the phonon–phonon scattering processes and thus decrease the thermal conductivity of the crystal. On the contrary, the quasi-localized character of the low-energy optical modes (dω_opt_/d*k* = 0) leads to very small phonon group velocities that further reduce the heat conductivity of these materials at low temperatures.^73^

Figure 4 displays the BP-like anomaly for a set of different materials, including ordered, disordered, and truly glass systems. The data are represented according to the scaled excess of heat capacity, [(*C*_*p*_ – *C*_D_)/*T*^3^]/[(*C*_*p*_ – *C*_D_)/*T*^3^]_max_, which is based on an analogous VDOS-scaled excess introduced by Malinovsky et al. some time ago.^74,75^ In Figure 4, one can clearly appreciate that the scaled excesses of the heat capacity of distinct types of solids (i.e., ordered and disordered crystals and structural glasses) are characterized by the same behavior to the near right and left of their maximum located at *T* = *T*_max_ (we neglect the TLS regime to the far left of the maximum). Such a generalized trend points toward a “universal” BP-like behavior of crystals, regardless of their degree of disorder.

**Figure 4:**
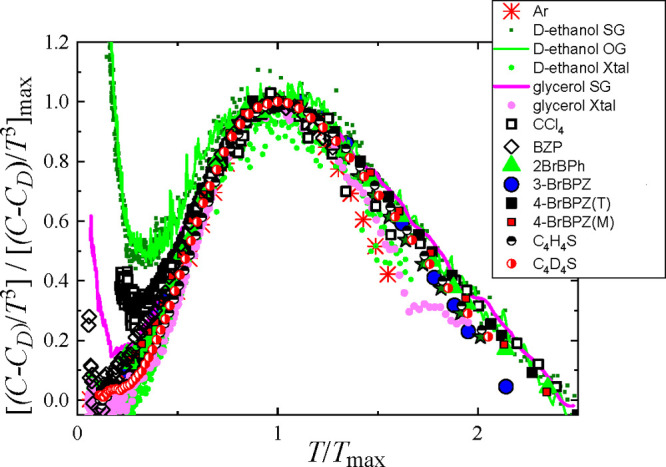
Experimental heat capacity data for benzophenone and its bromine-substituted derivatives (symbols as in Figure 1) expressed in the normalized coordinates [(*C*_*p*_ – *C*_D_)/*T*^3^]/[(*C*_*p*_ – *C*_D_)/*T*^3^]_max_ and as a function of *T*/*T*_max_. Literature data: Ar,^76^ red stars; deuterated ethanol^77^ for the orientational glass OG (dark green points), structural glass SG (light green line), and fully ordered crystal (light green points); glycerol,^78^ structural glass (pink line) and ordered crystal (pink points); deuterated (fully ordered, half-filled black circles) and normal (disordered, half-filled red circles) low-temperature phases of thiophene^16^ (filled green stars); CCl_4_, ordered monoclinic phase.^14^

In summary, the experimental BP-like anomalies reported for the low-temperature heat capacity of fully ordered BZP and several brominated derivatives represent compelling evidence that such an anomaly is not exclusive of glasses or, more generally, disordered solids. On the basis of the results of advanced first-principles calculations, we show that anharmonicity and the presence of low-energy quasi-localized optical modes play a central role in the emergence of the BP-like anomaly in fully ordered crystals.

## Supporting Information Available

Thermodynamic and structural details of the materials and details of the density functional theory calculations (PDF)
Transparent Peer Review report available (PDF)

## References

[ref1] Zeller R. C.; Pohl R. O. Thermal conductivity and specific heat of noncrystalline solids. Phys. Rev. B 1971, 4, 2029–2041. 10.1103/PhysRevB.4.2029.

[ref2] Phillips W. A. Amorphous Solids: Low Temperature Properties; Springer: Berlin, 1981.

[ref3] Pohl R. O.; Liu X.; Thompson E. J. Low-temperature thermal conductivity and acoustic attenuation in amorphous solids. E. J. Rev. Mod. Phys. 2002, 74, 991–1013. 10.1103/RevModPhys.74.991.

[ref4] Anderson P. W.; Halperin B. I.; Varma C. M. Anomalous low-temperature thermal properties of glasses and spin glasses. Philos. Mag. 1972, 25, 1–9. 10.1080/14786437208229210.

[ref5] Phillips W. A. Two-level states in glasses. Rep. Prog. Phys. 1987, 50, 1657–1708. 10.1088/0034-4885/50/12/003.

[ref6] Esquinazi P. Tunneling Systems in Amorphous and Crystalline Solids; Springer: Berlin, 1998.

[ref7] Buchenau U.; Galperin Y. M.; Gurevich V. L.; Schober H. R. Anharmonic potentials and vibrational localization in glasses. Phys. Rev. B 1991, 43, 5039–5045. 10.1103/PhysRevB.43.5039. 9997881

[ref8] Schirmacher W. Thermal conductivity of glassy materials and the “Boson peak”. Europhys. Lett. 2006, 73, 892–898. 10.1209/epl/i2005-10471-9.

[ref9] Milkus R.; Zaccone A. Local inversion-symmetry breaking controls the boson peak in glasses and crystals. Phys. Rev. B 2016, 93, 094204 10.1103/PhysRevB.93.094204.

[ref10] Chumakov A. I.; Monaco G.; Monaco A.; Crichton W. A.; Bosak A.; Ruffer R.; Meyer A.; Kargl F.; Comez L.; Fioretto D.; et al. Equivalence of the boson peak in glasses to the transverse acoustic van Hove singularity in crystals. Phys. Rev. Lett. 2011, 106, 225501 10.1103/PhysRevLett.106.225501. 21702612

[ref11] Grigera T. S.; Martín-Mayor V.; Parisi G.; Verrocchio P. Phonon interpretation of the ‘boson peak’in supercooled liquids. Nature 2003, 422, 289–292. 10.1038/nature01475. 12646916

[ref12] Shintani H.; Tanaka H. Universal link between the boson peak and transverse phonons in glass. Nat. Mater. 2008, 7, 870–877. 10.1038/nmat2293. 18849975

[ref13] Conyuh D. A.; Beltukov Y. M. Random matrix approach to the boson peak and Ioffe-Regel criterion in amorphous solids. Phys. Rev. B 2021, 103, 104204 10.1103/PhysRevB.103.104204. 34005859

[ref14] Moratalla M.; Gebbia J. F.; Ramos M. A.; Pardo L. C.; Mukhopadhyay S.; Rudíc S.; Fernandez-Alonso F.; Bermejo F. J.; Tamarit J. Ll. Emergence of glassy features in halomethane crystals. Phys. Rev. B 2019, 99, 024301 10.1103/PhysRevB.99.024301.

[ref15] Jeżowski A.; Strzhemechny M. A.; Krivchikov A. I.; Davydova N. A.; Szewczyk D.; Stepanian S. G.; Buravtseva L. M.; Romantsova O. O. Glassy anomalies in the heat capacity of an ordered 2-bromobenzophenone single crystal. Phys. Rev. B 2018, 97, 201201 10.1103/PhysRevB.97.201201.

[ref16] Miyazaki Y.; Nakano M.; Krivchikov A. I.; Koroyuk O. A.; Gebbia J. F.; Cazorla C.; Tamarit J. Ll. Low-temperature heat capacity anomalies in ordered and disordered phases of normal and deuterated thiophene. J. Phys. Chem. Lett. 2021, 12, 2112–2117. 10.1021/acs.jpclett.1c00289. 33625859 PMC8594864

[ref17] Gebbia J. F.; Ramos M. A.; Szewczyk D.; Jezowski A.; Krivchikov A. I.; Horbatenko Y. V.; Guidi T.; Bermejo F. J.; Tamarit J. Ll. Glassy Anomalies in the Low-temperature thermal properties of a minimally disordered crystalline solid. Phys. Rev. Lett. 2017, 119, 215506 10.1103/PhysRevLett.119.215506. 29219416

[ref18] Romanini M.; Negrier Ph.; Tamarit J. Ll.; Capaccioli S.; Barrio M.; Pardo L. C.; Mondieig D. Emergence of glassy-like dynamics in an orientationally ordered phase. Phys. Rev. B 2012, 85, 134201 10.1103/PhysRevB.85.134201.

[ref19] Szewczyk D.; Gebbia J. F.; Jeżowski A.; Krivchikov A. I.; Guidi T.; Cazorla C.; Tamarit J. Ll. Heat capacity anomalies of the molecular crystal 1-fluoro-adamantane at low temperatures. Sci. Rep. 2021, 11, 18640 10.1038/s41598-021-97973-2. 34545134 PMC8452677

[ref20] Christensen M.; Abrahamsen A. B.; Christensen N. B.; Juranyi F.; Andersen N. H.; Lefmann K.; Andreasson J.; Bahl Ch. R. H.; Iversen B. B. Avoided crossing of rattler modes in thermoelectric materials. Nat. Mater. 2008, 7, 811–815. 10.1038/nmat2273. 18758454

[ref21] Lanigan-Atkins T.; Yang S.; Niedziela J. L.; Bansal D.; May A. F.; Puretzky A. A.; Lin J. Y. Y.; Pajerowski D. M.; Hong T.; Chi S.; Ehlers G.; Delaire O. Extended anharmonic collapse of phonon dispersions in SnS and SnSe. Nat. Commun. 2020, 11, 4430 10.1038/s41467-020-18121-4. 32887880 PMC7474096

[ref22] Safarik D. J.; Schwarz R. B.; Hundley M. F. Similarities in the Cp/T^3^ Peaks in Amorphous and Crystalline Metals. Phys. Rev. Lett. 2006, 96, 195902 10.1103/PhysRevLett.96.195902. 16803111

[ref23] Reményi G.; Sahling S.; Biljaković K.; Starešinić D.; Lasjaunias J.-C.; Lorenzo J. E.; Monceau P.; Cano A. Incommensurate Systems as Model Compounds for Disorder Revealing Low-Temperature Glasslike Behavior. Phys. Rev. Lett. 2015, 114, 195502 10.1103/PhysRevLett.114.195502. 26024180

[ref24] Šimėnas M.; Balčiu̅nas S.; Svirskas Š.; Kinka M.; Ptak M.; Kalendra V.; Ga̧gor A.; Szewczyk D.; Sieradzki A.; Grigalaitis R.; et al. Phase diagram and cation dynamics of mixed MA_1–x_ FA_x_PbBr_3_ Hybrid Perovskites. Chem. Mater. 2021, 33, 5926–5934. 10.1021/acs.chemmater.1c00885.

[ref25] Ishii Y.; Ouchi Y.; Kawaguchi S.; Ishibashi H.; Kubota Y.; Mori S. Glassy anomalies in the lattice heat capacity of a crystalline solid caused by ferroelectric fluctuation. Phys. Rev. Mater. 2019, 3, 084414 10.1103/PhysRevMaterials.3.084414.

[ref26] Cano A.; Levanyuk A. P. Explanation of the Glasslike Anomaly in the Low-Temperature Specific Heat of Incommensurate Phases. Phys. Rev. Lett. 2004, 93, 245902 10.1103/PhysRevLett.93.245902. 15697828

[ref27] Li C. W.; Hellman O.; Ma J.; May A. F.; Cao H. B.; Chen X.; Christianson A. D.; Ehlers G.; Singh D. J.; Sales B. C.; Delaire O. Phonon self-energy and origin of anomalous neutron scattering spectra in snte and PbTe thermoelectrics. Phys. Rev. Lett. 2014, 112, 175501 10.1103/PhysRevLett.112.175501. 24836255

[ref28] Zhang H.; Wang X.; Chremos A.; Douglas J. F. Superionic UO_2_: A model anharmonic crystalline material. J. Chem. Phys. 2019, 150, 174506 10.1063/1.5091042. 31067868

[ref29] Babkov L. M.; Baran J.; Davydova N. A.; Drozd D.; Pyshkin O. S.; Uspenskiy K. E. Influence of the bromo group on the vibrational spectra and macroscopic properties of benzophenone derivatives. J. Mol. Struct. 2008, 887, 87–91. 10.1016/j.molstruc.2008.02.045.

[ref30] Fleischer E. B.; Sung N.; Hawkinson S. Crystal structure of benzophenone. J. Phys. Chem. 1968, 72, 4311–4312. 10.1021/j100858a065.

[ref31] Davydova N. A.; Mel’nik V. I.; Nelipovitch K. I.; Drozd M. Structural phase transitions and phosphorescence spectra in benzophenone. J. Mol. Struct. 2000, 555, 187–190. 10.1016/S0022-2860(00)00601-3.

[ref32] Hanaya M.; Hikima T.; Hatase M.; Oguni M. Low-temperature adiabatic calorimetry of salol and benzophenone and microscopic observation of their crystallization: finding of homogeneous-nucleation-based crystallization. J. Chem. Thermodyn. 2002, 34, 1173–1193. 10.1006/jcht.2002.0976.

[ref33] Chirico R. D.; Knipmeyer S. E.; Steele W. V. Heat capacities, enthalpy increments, and derived thermodynamic functions for benzophenone between the temperatures 5K and 440K. J. Chem. Thermodyn. 2002, 34, 1885–1895. 10.1016/S0021-9614(02)00261-6.

[ref34] Kutzke H.; Klapper H.; Hammond R. B.; Roberts K. J. Metastable β-phase of benzophenone: independent structure determinations via X-ray powder diffraction and single crystal studies. Acta. Crystallogr. B. Struct. 2000, 56, 486–496. 10.1107/S0108768100000355. 10877357

[ref35] Davydova N. A.; Mel’nik V. I.; Nelipovitch K.; Baran J.; Kukielski J. I. Heterogeneous structure of glassy benzophenone. Phys. Rev. B 2002, 65, 094201 10.1103/PhysRevB.65.094201.

[ref36] Romanini M.; Rietveld I. B.; Barrio M.; Negrier Ph.; Mondieig D.; Macovez R.; Céolin R.; Tamarit J. Ll. Uniaxial negative thermal expansion caused by π-π interactions in polymorphic 2-bromobenzophenone. Cryst. Growth Des. 2021, 21, 2167–2175. 10.1021/acs.cgd.0c01603.

[ref37] Baumer V. N.; Strzhemechny M. A.; Zloba D. I.; Zubatyuk R. I.; Romashkin R. V. Structure and phosphorescence of meta-bromobenzophenone crystal. J. Mol. Struct. 2012, 1021, 162–166. 10.1016/j.molstruc.2012.04.058.

[ref38] Zloba D. I.; Pyshkin O. S.; Buravtseva L. M.; Strzhemechny M. A. Phosphorescence of meta-brombenzophenone crystals over a wide temperature range. Low Temp. Phys. 2016, 42, 235–237. 10.1063/1.4943276.

[ref39] Ebbinghaus S.; Abeln D.; Epple M. Crystal structure of 4bromobenzophenone, Br-C_6_H_4_CO-C_6_H_5_ and 3, 4-dichlorobenzophenone, CI_2_-C_6_H_3_-CO-C_6_H_5_. Z. Kristallogr. - New Cryst. Struct. 1997, 212, 339–340.

[ref40] Strzhemechny M. A.; Baumer V. N.; Avdeenko A. A.; Pyshkin O. S.; Romashkin R. V.; Buravtseva L. M. Polymorphism of 4-bromobenzophenone. Acta Cryst. B 2007, 63, 296–302. 10.1107/S0108768106054334. 17374940

[ref41] Strzhemechny M. A.; Baumer V. N.; Avdeenko A. A.; Pyshkin O. S.; Romashkin R. V.; Buravtseva L. M. Polymorphism of 4-bromobenzophenone. Acta Crystallogr. B. Struct. Sci. Cryst. Eng. Mater. 2007, 63, 296–302. 10.1107/S0108768106054334. 17374940

[ref42] Pyshkin O. S.; Buravtseva L. M.; Baumer V. N.; Romashkin R. V.; Strzhemechny M. A.; Zloba D. I. Structure and low-temperature time-resolved phosphorescence spectra of crystalline and glassy 2-bromobenzophenone. Low Temp. Phys. 2009, 35, 580–588. 10.1063/1.3170935.

[ref43] Pässler R. Limiting Debye temperature behavior following from cryogenic heat capacity data for group-IV, III–V, and II–VI materials. Phys. Status Solidi B 2010, 247, 77–92. 10.1002/pssb.200945158.

[ref44] Baggioli M.; Zaccone A. Hydrodynamics of disordered marginally stale matter. Phys. Rev. Res. 2019, 1, 012010 10.1103/PhysRevResearch.1.012010.

[ref45] Cazorla C.; Boronat J. Simulation and understanding of atomic and molecular quantum crystals. Rev. Mod. Phys. 2017, 89, 035003 10.1103/RevModPhys.89.035003.

[ref46] Perdew J. P.; Ruzsinszky A.; Csonka G. I.; Vydrov O. A.; Scuseria G. E.; Constantin L. A.; Zhou X.; Burke K. Restoring the density-gradient expansion for exchange in solids and surfaces. Phys. Rev. Lett. 2008, 100, 136406 10.1103/PhysRevLett.100.136406. 18517979

[ref47] Kresse G.; Furthmuller J. Efficient iterative schemes for ab initio total-energy calculations using a plane-wave basis set. Phys. Rev. B 1996, 54, 11169 10.1103/PhysRevB.54.11169. 9984901

[ref48] Grimme S.; Antony J.; Ehrlich S.; Krieg H. A consistent and accurate ab initio parametrization of density functional dispersion correction (DFT-D) for the 94 elements H-Pu. J. Chem. Phys. 2010, 132, 154104 10.1063/1.3382344. 20423165

[ref49] Togo A.; Tanaka I. First principles phonon calculations in materials science. Scr. Mater. 2015, 108, 1–5. 10.1016/j.scriptamat.2015.07.021.

[ref50] Wei B.; Sun Q.; Li C.; Hong J. Phonon anharmonicity: a pertinent review of recent progress and perspective. Sci. China: Phys., Mech. Astron. 2021, 64, 117001 10.1007/s11433-021-1748-7.

[ref51] Van Hove L. The occurrence of singularities in the elastic frequency distribution of a crystal. Phys. Rev. 1953, 89, 1189–1193. 10.1103/PhysRev.89.1189.

[ref52] Kosevich A. M. Physical mechanics of real crystals; Naukova Dumka: Kiev, Ukraine, 1981.

[ref53] Baumert J.; Gutt C.; Shpakov V. P.; Tse J. S.; Krisch M.; Müller M.; Requardt H.; Klug D. D.; Janssen S.; Press W. Lattice dynamics of methane and xenon hydrate: Observation of symmetry-avoided crossing by experiment and theory. Phys. Rev. B 2003, 68, 174301 10.1103/PhysRevB.68.174301.

[ref54] Tse J. S.; Shpakov V. P.; Belosludov V. R.; Trouw F.; Handa Y. P.; Press W. Coupling of localized guest vibrations with the lattice modes in clathrate hydrates. EPL 2001, 54, 354–360. 10.1209/epl/i2001-00250-2.

[ref55] Christensen M.; Abrahamsen A. B.; Christensen N. B.; Juranyi F.; Andersen N. H.; Lefmann K.; Andreasson J.; Bahl C.R. H.; Iversen B. B. Avoided crossing of rattler modes in thermoelectric materials. Nat. Mater. 2008, 7, 811 10.1038/nmat2273. 18758454

[ref56] Takeuchi T.; Nagasako N.; Asahi R.; Mizutani U. Extremely small thermal conductivity of the Al-based Mackay-type 1/ 1-cubic approximants. Phys. Rev. B 2006, 74, 054206 10.1103/PhysRevB.74.054206.

[ref57] Tse J. S.; Li Z.; Uehara K. Phonon band structures and resonant scattering in Na_8_Si_46_ and Cs_8_Sn_44_ clathrates. EPL 2001, 56, 261–267. 10.1209/epl/i2001-00515-8.

[ref58] Samanta M.; Pal K.; Pal P.; Waghmare U. V.; Biswas K. Localized vibrations of Bi bilayer leading to ultralow lattice thermal conductivity and high thermoelectric performance in weak topological insulator n-type BiSe. J. Am. Chem. Soc. 2018, 140, 5866–5872. 10.1021/jacs.8b02691. 29641193

[ref59] Lee W.; Li H.; Wong A. B.; Zhang D.; Lai M.; Yu Y.; Kong Q.; Lin E.; Urban J. J.; Grossman J. C.; Yang P. Ultralow thermal conductivity in all-inorganic halide perovskites. Proc. Natl. Acad. Sci. U.S.A. 2017, 114, 8693–8697. 10.1073/pnas.1711744114. 28760988 PMC5565476

[ref60] Zhang Z.; Hu S.; Nakayama T.; Chen J.; Li B. Reducing lattice thermal conductivity in schwarzites via engineering the hybridized phonon modes. Carbon 2018, 139, 289–298. 10.1016/j.carbon.2018.06.057.

[ref61] Bouyrie Y.; Candolfi C.; Pailhès S.; Koza M. M.; Malaman B.; Dauscher A.; Tobola J.; Boisron O.; Saviot L.; Lenoir B. From crystal to glass-like thermal conductivity in crystalline minerals. Phys. Chem. Chem. Phys. 2015, 17, 19751–19758. 10.1039/C5CP02900G. 26109211

[ref62] Baggioli M.; Cui B.; Zaccone A. Theory of the phonon spectrum in host-guest crystalline solids with avoided crossing. Phys. Rev. B 2019, 100, 220201 10.1103/PhysRevB.100.220201.

[ref63] Dong Z. Y.; Zhou Y.; Chen X. Q.; Li W. J.; Cao Z. Y.; Luo C.; Zhong G. H.; Peng Q.; Wu X.; Chen X. J. Effect of low-frequency optical phonons on the thermal conductivity of 2H molybdenum disulfide. Phys. Rev. B 2022, 105, 184301 10.1103/PhysRevB.105.184301.

[ref64] Yang Z. Y.; Wang Y. J.; Zaccone A. Correlation between vibrational anomalies and emergent anharmonicity of the local potential energy landscape in metallic glasses. Phys. Rev. B 2022, 105, 014204 10.1103/PhysRevB.105.014204.

[ref65] Duval E.; Mermet A.; Saviot L. Boson peak and hybridization of acoustic modes with vibrations of nanometric heterogeneities in glasses. Phys. Rev. B 2007, 75, 024201 10.1103/PhysRevB.75.024201.

[ref66] Klinger M. L.; Kosevich A. M. Soft-mode-dynamics model of acoustic-like high-frequency excitations in boson-peak spectra of glasses. Phys. Lett. A 2001, 280, 365–370. 10.1016/S0375-9601(01)00090-1.

[ref67] Tomoshige N.; Goto S.; Mizuno H.; Mori T.; Kim K.; Matubayasi N. Understanding the scaling of boson peak through insensitivity of elastic heterogeneity to bending rigidity in polymer glasses. J. Phys.: Condens. Matter 2021, 33, 274002 10.1088/1361-648X/abfd51. 33930889

[ref68] Ren S.; Zong H.-X.; Tao X.-F.; Sun Y.-H.; Sun B. A.; Xue D.-Z.; Ding X.-D.; Wang W.-H. Boson-peak-like anomaly caused by transverse phonon softening in strain glass. Nat. Commun. 2021, 12, 5755 10.1038/s41467-021-26029-w. 34599172 PMC8486772

[ref69] Rufflé B.; Parshin D. A.; Courtens E.; Vacher R. Boson Peak and its Relation to Acoustic Attenuation in Glasses. Phys. Rev. Lett. 2008, 100, 015501 10.1103/PhysRevLett.100.015501. 18232782

[ref70] Baggioli M.; Zaccone A. Universal origin of boson peak vibrational anomalies in ordered crystals and in amorphous materials. Phys. Rev. Lett. 2019, 122, 145501 10.1103/PhysRevLett.122.145501. 31050477

[ref71] Baggioli M.; Zaccone A. Low-energy optical phonons induce glassy-like vibrational and thermal anomalies in ordered crystals. JPhys Mater. 2020, 3, 015004 10.1088/2515-7639/ab4758.

[ref72] Romantsova O. O.; Horbatenko Yu. V.; Krivchikov A. I.; Korolyuk O. A.; Vdovichenko G. A.; Zloba D. I.; Pyshkin O. S. Anomalous heat transfer in two polymorphs of para-bromobenzophenone. Low Temp. Phys. 2017, 43, 395–399. 10.1063/1.4979956.

[ref73] Qian X.; Zhou J.; Chen G. Phonon-engineered extreme thermal conductivity materials. Nat. Mater. 2021, 20, 1188 10.1038/s41563-021-00918-3. 33686278

[ref74] Malinovsky V. K.; Novikov V. N.; Parshin P.; Sokolov A. P.; Zemlyanov M. G. Universal form of the low-energy (2 to 10 meV) vibrational spectrum of glasses. Europhys. Lett. 1990, 11, 43–47. 10.1209/0295-5075/11/1/008.

[ref75] Malinovsky V. K.; Novikov V. N.; Sokolov A. P. Log-normal spectrum of low-energy excitations in glasses. Physics Lett. A 1991, 153, 63–69. 10.1016/0375-9601(91)90363-D.

[ref76] Bagatskii M. I.; Krivchikov A. I.; Manzhelii V. G. Calorimetric studies of rotational motion of ^14^n_2_ molecules in solid argon matrix. Fiz. Nizk. Temp. 1987, 13, 423–429.

[ref77] Talón C.; Ramos M. A.; Vieira S. Low-temperature specific heat of amorphous, orientational glass, and crystal phases of ethanol. Phys. Rev. B 2002, 66, 012201 10.1103/PhysRevB.66.012201.

[ref78] Talón C.; Zou Q. W.; Ramos M. A.; Villar R.; Vieira S. Low-temperature specific heat and thermal conductivity of glycerol. Phys. Rev. B 2001, 65, 012203 10.1103/PhysRevB.65.012203.

