# Supplementary material for: Role of Optical Phonons and Anharmonicity in the Appearance of the Heat Capacity Boson Peak-like Anomaly in Fully Ordered Molecular Crystals
Source: J Phys Chem Lett. 2022 Jun 2;13(22):5061–7. doi: 10.1021/acs.jpclett.2c01224 (PMC9189925; doi:10.1021/acs.jpclett.2c01224)
Supplement: Supplementary file 1 — jz2c01224_si_001.pdf [file jz2c01224_si_001.pdf]

Supplemental Material for:
The Role of Optical Phonons and Anharmonicity in the Appearance of the Heat Capacity Boson Peak-Like Anomaly in Fully Ordered Molecular Crystals

Alexander I. Krivchikov[1], Andrzej Jeżowski[2], Daria Szewczyk[2], Oxsana A. Korolyuk[1], Olesya O. Romantsova[1], Lubov M. Buravtseva[1], Claudio Cazorla[3] and Josep Ll. Tamarit[3,*]

[1]B. Verkin Institute for Low Temperature Physics and Engineering of the National Academy of Sciences of Ukraine, 47 Nauky Ave., Kharkiv, 61103, Ukraine

[2]Institute of Low Temperature and Structure Research, Polish Academy of Sciences, 2 Okólna Str., 50-422 Wrocław, Poland

[3] Grup de Caracterizació de Materials, Departament de Fisica, EEBE and Barcelona Research Center in Multiscale Science and Engineering, Universitat Politècnica de Catalunya, Eduard Maristany, 10-14, 08019 Barcelona, Catalonia, Spain

Materials

Benzophenones are a group of so-called flexible molecules. If only one hydrogen atom is replaced by a halogen atom (F, Cl, Br, I), a significant change in the structure and energy of the molecule occurs. Bromobenzophenones $C_{13}H_9BrO$ can form isomers that differ in the position of the bromine atom in the phenyl ring: 2-, 3- and 4-bromobenzophenone, or *ortho*-, *meta*- and *para*-bromobenzophenone, respectively. In 2, 3- and 4-bromobenzophenone, the heavy bromine atom replaces the hydrogen atom, which is located relative to the carbonyl group C=O in the 2nd, 3rd or 4th position, respectively. The position of the bromine atom in the phenyl ring strongly changes the geometry of the molecule, which leads to a change in the physical properties of bromobenzophenones, such as the melting point temperature and the ability to form glass[1].

The properties of the molecular crystal of unsubstituted benzophenone $(C_6H_5)_2CO$ (hereinafter referred to as **BZP**) have been actively studied both theoretically and experimentally: eg. by the method of three-dimensional X-ray diffraction[2], Raman[4,5] spectroscopy and differential scanning calorimetry, see, eg., adiabatic calorimetry[6,7], X-ray powder diffraction[8], photoluminescence[0]; there are also thermal conductivity data available[10]. The **BZP** molecule has the shape of a C_2 symmetric butterfly: the carbonyl group is bonded to two phenyl rings. The chemical bond between carbon and oxygen atoms in the carbonyl group is double. The planes in which the rings lie form a large

dihedral angle. According to the structural data, the **BZP** molecular crystal displays polymorphism [8, 11], the fully ordered stable α phase is orthorhombic ($P2_12_12_1$)[2,11], while the fully ordered metastable β phase is monoclinic ($C2/c$)[12]. The unit cell of the α phase of benzophenone contains 4 molecules (Z = 4)[8]; melting point T_m = 323.9 K [4]. Upon very rapid cooling, liquid benzophenone can be obtained in the molecular glass state[3] below the glass transition temperature, $T_g \approx 211.7$ K. When heated, the glass passes into a supercooled liquid, and then into the β phase at $T \approx 250–263$ K [4]; in the work of ref. 13, the transition temperature T = 243 K was found. The β → α transition is irreversible and proceeds easily at 275–285 K [13]. In the present work, we investigated the heat capacity of the α phase of **BZP** in the temperature range 0.39 to 9 K.

Due to the presence of a heavy bromine atom, the shape of the 2-bromobenzophenone molecule (hereinafter referred to as **2-**BrBZP) in the molecular crystal is characterized by a strong asymmetry of the torsion angles of the two phenyl rings, see, *eg.*, refs. 1 and 14. In optical studies of the phosphorescence of the **2-**BrBZP crystal[15], the conformational parameters of the molecule were studied, and it was shown that they strongly change with temperature, due to which **2-**BrBZP shows unusual properties in the condensed state. In the stable crystalline phase **2-**BrBP has a monoclinic $P2_1/a$ structure, Z = 4 [15,16]. The melting point obtained by differential scanning calorimetry upon heating the crystal is 318 K [1], the work or ref. 17 indicated that temperature is 316.5 K. **2-**BrBZP shows very unusual resistance to crystallization[1,1,5,18]. It was found that the cooling of the liquid phase leads to a distinct glass transition process, which begins (onset) at T_g = 225.1 K[1]. Recently, by the methods of infrared spectroscopy and differential scanning calorimetry (FT-IR spectroscopy and DSC), the emergence of a new polymorphic metastable phase was discovered, which was formed at a temperature of 245 K during the heating of the glass state[17]. The structure of the metastable phase, which melts at 301 K and passes into the stable phase at room temperature[17], has been recently reported[19]. Crystal **2-**BrBZP has a low-frequency optical branch in its spectrum

It is known that molecular 3-bromobenzophenone (hereinafter referred to as **3-**BrBZP) melts at 81 °C as described in CRC[20,21]. **3-**BrBZP crystals were investigated using X-ray diffraction, integrated phosphorescence spectrometry and *ab initio* calculations. It was shown that crystalline **3-**BrBZP has an orthorhombic *Pbca* structure, Z = 8[20]. Anomalies were found in the phosphorescence spectra of **3-**BrBZP crystals, which may be a consequence of the structural features of both the molecule itself and the crystal[22].

The properties of the molecular crystal of 4-bromobenzophenone (hereinafter referred to as **4-**BrBZP) have been studied quite actively: there are structural data [23,24], differential scanning calorimetry data, IR and Raman vibrational spectra[1], phosphorescence spectra[25], as well as data on thermal conductivity[26,27]. Crystalline **4-**BrBZP exhibits polymorphism. Studies using X-ray diffractometry[25] showed that the stable polymorph has a monoclinic $P2_1/c$ structure, Z = 4, T_m = 355.2 K, and the metastable polymorph has a triclinic $P\bar{1}$ structure, Z = 2, T_m = 354.0 K. The peculiarity of these two polymorphic modifications is that in the triclinic polymorph in the chain of molecules there is an alternation of molecules one after another, while in the monoclinic polymorph there is an alternation of pairs of molecules[24,25].

Preparation of samples

The benzophenone source material was of the grade "technically pure" (Shostka chemical reagents factory, former USSR). The original material was subjected to purification using the zone melting procedure. The rate of progress of the ampoule during that process was 10 mm per hour; the number of zone melting passes was about 50. Cool-off of the areas above and under the melting zone was provided with room-temperature water flow cooled shields. Purity of the final **BZP** material was checked by phosphorescence spectra. A **BZP** single crystal has been grown in a glass ampoule (pumped down to 10^{-3} Torr) with narrowed end where crystal seeds emerged and grew. In order to obtain single crystal, the standard (Bridgman) procedure was employed: the ampoule with the melt was slowly (at a rate about 0.5 mm/h) driven through a furnace with stabilized temperature gradient. The newly grown crystal was submitted to an additional annealing. The crystal, grown according to the above procedure, belong to the stable α modification.

To grow a **2-**BrBZP single crystal, we took the starting material of the "pure" (Shostka chemical reagents factory, former USSR). For additional purification of the substance, the method of multiple recrystallization from ethanol solution was used. Crystals grew during 5–6 days. The resulting single crystals had typical dimensions 1×5×8 mm^3. Check phosphorescence measurements showed very low concentrations of lattice defects. Larger single crystals (10×10×15 mm^3) were grown from seeds in a supercooled phase according to the known procedure[28]. A transparent, colorless single crystal with a nice cut was chosen to measure the heat capacity.

The starting material for the preparation of a sample of stable polymorph **4-**BrBZP was a "pure" (L'vov chemical reagents factory, former USSR). Additionally, the substance was purified by the method of gradient sublimation. **4-**BrBZP obtained by sublimation had a monoclinic structure $P2_1/c$ and was a mechanical mixture of small needle-shaped crystallites with typical sizes (0.2–0.3)×(0.3–0.5)×(0.5–1) mm^3. To obtain a bulk sample, this **4-**BrBZP powder substance was pressed in several stages, during which the powder was admitted into the container in portions and compacted. A sample of the metastable polymorph **4-**BrBZP was obtained by the Bridgman method and consisted of a single crystal of the triclinic structure [24] with the size of 5×3×2 mm^3.

To obtain **3-**BrBZP samples, we used Aldrich material in the form of a powder with purity of 98 %, which was subjected to two successive sublimation purifications by the method of gradient sublimation. The purification method was the same as for **4-**BrBZP. The samples obtained as a result of sublimation were polycrystals with an average size of 0.5×0.5×0.5 mm^3, had a generally globular shape without a clear edge. A bulk polycrystalline sample was obtained by pressing, similar to the sample of stable polymorph **4-**BrBZP. Attempts to grow a bulk crystal by the Bridgman method or from a solution with various solvents did not give a positive result.

Experimental methods

Heat capacity *Cp* measurements of 2-BrBZP, 3-BrBZP and two 4-BrBZP polymorphs were carried out using the physical property measurement system (PPMS) manufactured by Quantum Design Inc. with the option for measuring heat capacity

(relaxation-type calorimeter). A detailed description of the setup and measurement process is given in ref. 29. Despite the fact that the experiments were carried out in the temperature range 1.8–300 K, a narrower region of low temperatures is of interest for discussion henceforth. Measurements of the heat capacity *Cp* of BP at temperatures of 0.39–9 K were also carried out using the system (PPMS, Quantum Design) equipped with a 3He refrigeration option[29].

Computational methods. First-principles calculations based on density functional theory (DFT)

Density functional theory (DFT) calculations[30] based on the PBE functional[31] were performed with the VASP software[32]. Long-ranged dispersion interactions were captured with the DFT-D3 method[33]. Wave functions were represented in a plane-wave basis truncated at 650 eV and a Monkhorst–Pack k-point grid of 2 × 2 × 4 (2 × 4 × 4) was employed for integrations within the first Brillouin zone of the stable BZP and 4-BrBZP phases (metastable 4-BrBZP phase). Phonon calculations were performed within the harmonic approximation by means of density functional perturbation theory calculations (□ point) [32] and the small displacement method (full phonon spectrum) [34]. Heat capacities were subsequently estimated within the quasi-harmonic approximation by using dense □-centered k-point meshes of 16 × 16 × 16 for sampling of the first Brillouin zone[30,34].

In order to get rid of the vibrational phonon instabilities appearing in the phonon spectra of the analyzed molecular crystals at normal pressure, we considered an external hydrostatic pressure of ≈2 GPa in our calculations. The only expected physical changes deriving from such a pressure-induced vibrational stabilization are a generalized increase in the vibrational energy levels (e.g., the first optical □ phonon mode in 4-BrBZP(T) moves from 4.31 at normal pressure to 6.75 meV at ≈2 GPa) and a consequent upwards shift in the characteristic temperatures Θ_D (i.e., the Debye temperature) and $T_{\max}$ (i.e., the temperature at which the Boson-like heat capacity anomaly appears).

Specifically, based on the outcomes of elastic-constant DFT calculations we estimated a Debye temperature of 392 and 425 K for pressurized 4-BrBZP(T) and 4-BrBZP(M), respectively, while the corresponding experimental values are 80 and 82 K. Thus, at the qualitative level, the agreement between our experiments and calculations is satisfactory in the sense that we theoretically reproduce the fundamental Θ_D differences among the two 4-BrBZP polymorphs. Likewise, we computed a $T_{\max}$ of 12.2 (+/-0.5) and 13.5(+/-0.5) K for pressurized 4-BrBZP(T) and 4-BrBZP(M), respectively (see inset in the Supplementary Figure S1), while the corresponding experimental values are 6.7 (+/-0.3) and 6.9 (+/-0.3) K. Moreover, in our DFT calculations we find that in agreement with the experiments the C/T^3 maximum is slightly larger in 4-BrBZP(T) than in 4-BrBZP(M) (see Supplementary **Fig.S1**), and in the scaled $C/T^3/(C/T^3)^{\max}$ vs. $T/T^{\max}$ representation the agreement between our calculations and the experiments can be considered as practically quantitative.

Thus, overall the qualitative agreement between our experiments and DFT calculations for the vibrational and heat capacity properties of the analyzed molecular crystals can be regarded as highly satisfactory (see Supplementary **Fig.S1**)

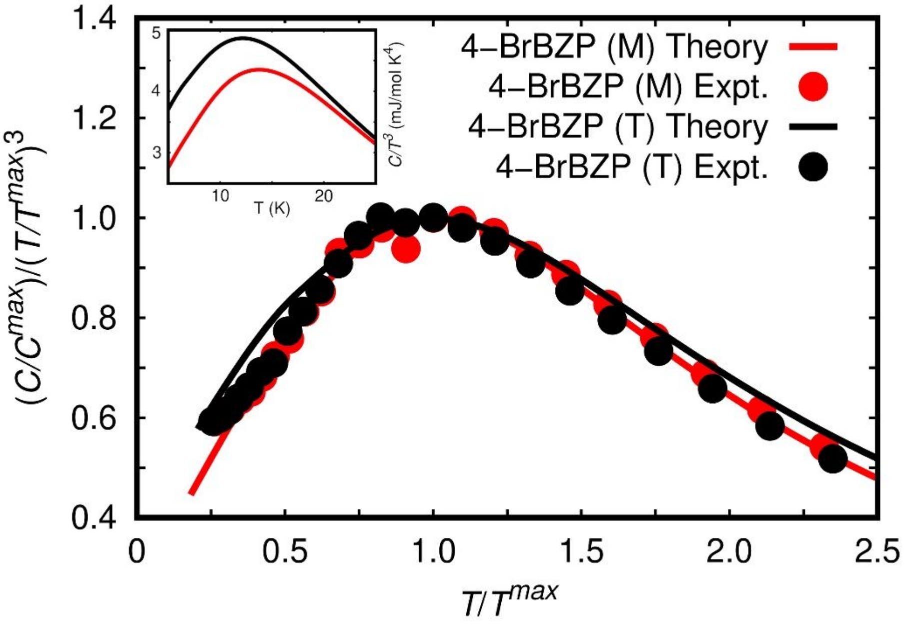

Supplementary Fig.S1: Calculated heat capacity of pressurized 4-BrBZP(M) and 4-BrBZP(T). In the scaled $C/T^3/(C/T^3)^{\max}$ vs. $T/T^{\max}$ representation, the quantitative agreement between the DFT calculations (solid lines) and experiments (solid points) is very good. In the non-scaled C/T^3 vs. T representation (inset), the agreement between the experiments and DFT calculations is not quantitative due to the artificial pressurization considered in the calculations; however, the qualitative agreement between the experiments and DFT calculations is fairly good (i.e., the Boson peak-like is larger for 4-BrBZP(T) than for 4-BrBZP(M) and the temperatures at which they appear in both systems are very similar).

References

(1) Babkov, L. M.; Baran, J.; Davydova, N. A.; Drozd, D.; Pyshkin, O. S.; Uspenskiy, K. E. Influence of the bromo group on the vibrational spectra and macroscopic properties of benzophenone derivatives. *J. Mol. Struct.* **2008**, *887*, 87–91.

(2) Fleischer, E. B.; Sung, N.; Hawkinson, S. Crystal structure of benzophenone. *J. Phys. Chem.* **1968,** *72*, 4311–4312.

(3) Davydova, N. A.; Mel'nik, V. I.; Nelipovitch, K. I.; Drozd, M. Structural phase transitions and phosphorescence spectra in benzophenone. *J. Mol. Struct.* **2000,** *555,* 187–190.

(4) Davydova, N. A.; Mel'nik, V. I.; Nelipovitch, K. I.; Baran, J. Low-frequency Raman scattering from glassy and supercooled liquid benzophenone. *J. Mol. Struct.* **2001,** *563*, 105–109.

(5) Davydova, N. A.; Mel'nik, V. I.; Nelipovich, K. I.; Baran, Y.; Drozd, M. The boson peak and nanostructure of vitreous benzophenone. *Solid State Phys*. **2001**, *43,* 1589–1593.
(6) Hanaya, M.; Hikima, T.; Hatase, M.; Oguni, M. Low-temperature adiabatic calorimetry of salol and benzophenone and microscopic observation of their crystallization: finding of homogeneous-nucleation-based crystallization. *J. Chem. Thermodyn*. **2002**, *34*, 1173–1193.
(7) Chirico, R. D.; Knipmeyer, S. E.; Steele, W. V. Heat capacities, enthalpy increments, and derived thermodynamic functions for benzophenone between the temperatures 5K and 440K. *J. Chem. Thermodyn*. **2002,** *34*, 1885–1895.
(8) Kutzke, H.; Klapper, H.; Hammond, R. B.; Roberts, K. J. Metastable β-phase of benzophenone: independent structure determinations via X-ray powder diffraction and single crystal studies. *Acta Cryst B* **2000**, *56*, 486–496.
(9) Davydova, N. A.; Mel'nik, V. I.; Nelipovitch, K.; Baran, J.; Kukielski, J. I. Heterogeneous structure of glassy benzophenone. *Phys. Rev. B* **2002, *65*,** 094201.
(10) Jeżowski, A.; Strzhemechny, M. A.; Krivchikov, A. I.; Pyshkin, O. S.; Romantsova, O. O.; Korolyuk, O. A.; Filatova, A. Thermoactivated heat transfer mechanism in molecular crystals: Thermal conductivity of benzophenone single crystals. *AIP Advances* **2019,** *9*, 015121.
(11) Lobanova, G. M. Molecular and crystal structure of benzophenone. *Soviet Physics Crystallography,* **1968,** *13*, 856–858.
(12) Kutzke, H.; Klapper, H.; Hammond, R. B.; Roberts, K. J. Metastable β-phase of benzophenone: independent structure determinations via X-ray powder diffraction and single crystal studies. *Acta Cryst. B* **2000**, *56*, 486–496.
(13) Babkov, L.; Baran, J.; Davydova, N. A.; Mel'nik, V. I.; Uspenskiy, K. E. Raman spectra of metastable phase of benzophenone. *J. Mol. Struct*. **2006,** *792*, 73–77.
(14) Avdeenko, A. A.; Pyshkin, O. S.; Eremenko, V. V.; Strzhemechny, M. A.; Buravtseva, L. M.; Romashkin, R. V. Photoluminescence of ortho-bromobenzophenone. *Low Temp. Phys*. **2006,** *32*, 1028–1034.
(15) Pyshkin, O. S.; Buravtseva, L. M.; Baumer, V. N.; Romashkin, R. V.; Strzhemechny, M. A.; Zloba D. I. Structure and low-temperature time-resolved phosphorescence spectra of crystalline and glassy 2-bromobenzophenone. *Low Temp. Phys*. **2009,** *35*, 580–588.
(16) Baumer, V. N.; Romashkin, R. V.; Strzhemechny, M. A.; Avdeenko, A. A.; Pyshkin, O. S.; Zubatyuk, R. I.; Buravtseva, L. M. 2-Bromobenzophenone. *Acta Crystallographica Section E: Structure Reports Online*, **2005,** *61*, o1170–o1172.
(17) Baran, J.; Davydova, N. A.; Drozd, M. Discovery of a new polymorphic phase of ortho-bromobenzophenone. *Chem. Phys. Lett.* **2015**, *621*, 18–21.
(18) Buravtseva, L. M.; Pyshkin, O. S.; Strzhemechny, M. A.; Avdeenko, A. A. Phosphorescence of vitreous 2-bromobenzophenone. *Low Temp. Phys*. **2008,** *34*, 466–469.
(19) Romanini, M.; Rietveld, I. B.; Barrio, M.; Negrier, Ph.; Mondieig, D.; Macovez, R.; Céolin, R.; Tamarit, J. Ll. Uniaxial negative thermal expansion caused by π-π interactions in polymorphic 2-bromobenzophenone. *Cryst. Growth Des.* **2021,** *21*, 2167–2175.
(20) Weast, R. C. *CRC Handbook of Chemistry and Physics*, *66th ed*.; CRC Press, Boca Raton, 1986.

(21) Baumer, V. N.; Strzhemechny, M. A.; Zloba, D. I.; Zubatyuk, R. I.; Romashkin, R. V. Structure and phosphorescence of meta-bromobenzophenone crystal. *J. Molec. Struct.,* **2012,** *1021*, 162–166.
(22) Zloba, D. I.; Pyshkin, O. S.; Buravtseva L. M.; Strzhemechny, M. A. Phosphorescence of meta-brombenzophenone crystals over a wide temperature range. *Low Temp. Phys*. **2016**, *42*, 235–237.
(23) Ebbinghaus, S.; Abeln, D.; Epple, M. Crystal structure of 4-bromobenzophenone, Br-C_6H_4CO-C_6H_5 and 3, 4-dichlorobenzophenone, CI_2-C_6H_3-CO-C_6H_5. *Z Krist-New Cryst*. **1997,** *212*, 339–340.
(24) Strzhemechny, M. A.; Baumer, V. N.; Avdeenko, A. A.; Pyshkin, O. S.; Romashkin R. V.; Buravtseva, L. M. Polymorphism of 4-bromobenzophenone. *Acta Cryst. B* **2007,** *63*, 296–302.
(25) Zloba, D. I.; Buravtseva, L. M.; Pyshkin, O. S.; Strzhemechny, M. A. Phosphorescence of the polymorphs of 4-bromobenzophenone. *Low Temp. Phys*. **2013,** *39,* 1103–1109.
(26) Strzhemechny, M. A.; Krivchikov, A. I.; Jeżowski, A.; Zloba, D. I.; Buravtseva, L. M.; Churiukova, O.; Horbatenko, Yu. V. New thermal conductivity mechanism in triclinic 4-bromobenzophenone crystal. *Chem. Phys. Lett*. **2016,** *647*, 55–58.
(27) Romantsova, O. O.; Horbatenko, Y. V.; Krivchikov, A. I.; Korolyuk, O. A.; Vdovichenko, G. A.; Zloba, D. I.; Pyshkin, O. S. Anomalous heat transfer in two polymorphs of para-bromobenzophenone. *Low Temp. Phys*. **2017,** *43*, 395–399.
(28) Wang, W. L.; Wang, M.; Huang, W. D. A novel growth method for organic nonlinear optical crystals. *Opt. Mater*. **2004,** *27*, 609–612.
(29) Szewczyk D.; Jeżowski, A.; Krivchikov, A. I.; Tamarit, J. Ll. Influence of thermal treatment on thermal properties of adamantane derivatives. *Low Temp. Phys*. **2015,** *41*, 469–472.
(30) Cazorla, C.; Boronat, J. Simulation and understanding of atomic and molecular quantum crystals. *Rev. Mod. Phys*. **2017,** *89*, 035003.
(31) Perdew, J. P.; Ruzsinszky, A.; Csonka, G.I.; Vydrov, O. A.; Scuseria, G. E.; Constantin, L. A.; Zhou, X.; Burke K. Restoring the density-gradient expansion for exchange in solids and surfaces. *Phys. Rev. Lett*. **2008**, *100,* 136406.
(32) Kresse, G.; Furthmuller, J. Efficient iterative schemes for ab initio total-energy calculations using a plane-wave basis set. *Phys. Rev. B* **1996,** *54*, 11169.
(33) Grimme, S.; Antony, J.; Ehrlich, S.; Krieg, H. A consistent and accurate ab initio parametrization of density functional dispersion correction (DFT-D) for the 94 elements H-Pu. *J. Chem. Phys.* **2010**, *132*, 154104.
(34) Togo, A.; Tanaka, I. First principles phonon calculations in materials science. *Scr. Mater*. **2015,** *108*, 1.
