# Supplementary material for: Role of Optical Phonons and Anharmonicity in the Appearance of the Heat Capacity Boson Peak-like Anomaly in Fully Ordered Molecular Crystals
Source: J Phys Chem Lett. 2022 Jun 2;13(22):5061–7. doi: 10.1021/acs.jpclett.2c01224 (PMC9189925; doi:10.1021/acs.jpclett.2c01224)
Supplement: Supplementary file 2 — jz2c01224_si_002.pdf [file jz2c01224_si_002.pdf]

jz-2022-01224e.R1

Name: Peer Review Information for "The Role of Optical Phonons and Anharmonicity in the Appearance of the Heat Capacity Boson Peak-Like Anomaly in Fully Ordered Molecular Crystals"

First Round of Reviewer Comments

Reviewer: 1

Comments to the Author

This is a beautiful paper elucidating the origin of the boson peak through a combination of careful measurement in conjunction with cutting edge simulations for a class of model crystalline materials for which a the boson peak has been observed in recent measurements. The simulations show that the boson peak in these materials arise from a mode coupling phenomena between the optical and acoustic modes, a coupling that has also been implicated elsewhere in impacting the dispersion relation of the dynamic structure of glass-forming liquids so the effect described is probably of broader significance to the thermodynamics and dynamics of condensed materials. The findings also accord with recent theoretical predictions of Bagglioli and Zaccone (refs. 67 and 67) and suggestions made in some earlier studies. The paper is important because it suggests this mechanism, which was formerly somewhat controversial, in a highly convincing fashion. I strongly endorse the publication of this paper in J. Chem. Phys. Lett.

The only significant criticism that I would make relates to the last sentence of the paper's abstract. which is potentially misleading and which could be simply deleted.

There are many physical effects that lead anharmonicity in condensed materials and there is correspondingly no simple unique parameter quantifying the "degree of anharmonicity". The main finding of the paper is that the boson peak can arise purely from anharmonic interactions so structural disorder is not required. It is a separate question of measure or measures of anharmonicity, or properties sensitive to this anharmonicity, determine the height of the boson peak as well as the tempearture dependence of the boson peak frequency. The authors should thus delete their overly broad statement at the end of their paper.

As an optional matter, i note that the boson peak phenomenon has been noted ibefore in simulations of anharmonic crystalline materials and that such materials are becoming of extreme interest recently in connection of energy conversion, battery and other energy related applications and scientific studies in the contaxts of geophysical and astrophysical phenomena. In particular, superionic materials appear to be gaining a lot of recent interest along this lines and i mention a recent molecular dynamics simulation study of the superionic dynamics of crystalline UO2, a major component of reactor fuel, where the boson peak was observed under superionic conditions. Again there is a broader sphere of problems that the authors results have relevance for that might be mentioned briefly through some referencing. This is left as an option.

Reviewer: 2

Comments to the Author

The work presented by Krivchikov and co-workers provides an interesting and fundamentally important study. The study is based on data for crystals, which are used to discuss and elucidate the origin of the so-called “Boson peak”, which has for long time been associated with amorphous or partly disordered materials. Based on the results the authors suggest origins of the peak that are unrelated to disorder and corroborate recent theory regarding the Boson peak. The results are important for a deeper understanding of the vibrational density of states in general and the Boson peak in particular. Eventually, this can lead to a more refined theory for heat capacity, and other properties related to the vibrational density of state, beyond that of the Debye theory.

Minor issues:

1. Perhaps the authors mean “piling-up” of low-frequency modes instead of “pilling-up”.

2. I think the comparison of the boson peak between materials with different order is interesting and relevant – hard to understand why it is in the supplementary information. If it is due to length restrictions, then I think it is more interesting than Fig 2, but this assessment is of course up to the authors. In this context, it could also be interesting to include data for amorphous/glassy “BZP”, which can be obtained by rapid cooling (supplementary information). In that case, a non-normalized plot would also be interesting.

3. Supplementary information: There might be a mistake in the description of the source of the materials. It is claimed that some materials was purchased in “USSR”, which vanished in 1991.

4. Although the authors find quantitative agreement between the results of the DFT calculations and experimental data, the results seem to imply a rather large Gruneisen parameter. For example, the (large) increase of the Debye temperature from the experimental value at 1 atm of 82 K to 425 K calculated at 2 GPa indicates a larger Gruneisen parameter than normally found for crystals.

Reviewer: 3

Comments to the Author

I have read this manuscript with much interest. The authors present a compelling experimental and computational study revealing the origin of glasslike anomalies in perfectly ordered anharmonic crystals. The boson peak observed in both VDOS and specific heat is convincingly attributed to either piling up of low-lying optical modes or pseudo-van Hove peaks due to leveling off at avoided crossing between acoustic and low optical modes. This mechanism is going to play an important role for our physico-chemical understanding of glasslike properties in perfectly ordered crystals and for energy materials such as thermoelectrics. I recommend publication of the manuscript with only minor revisions listed below. This is groundbreaking work that certainly belongs to JPCL and will have a great impact on chemistry, physics, and materials science.

- The authors mention that the DFT method is only harmonic. However, from the simulated snapshots of the molecular systems it should be possible to infer also quantitative measures of anharmonicity, see e.g. Phys. Rev. B 105, 184301 (2022) or Phys. Rev. B 105, 014204 (2022). It could be nice, if possible, if the authors could add some measure of anharmonicity in their simulations, especially for the phonons that give rise to the boson peak. Ultimately, the reason why the optical phonons are so low is the anharmonicity (just think of damped harmonic oscillator, when you increase the damping the frequency of the oscillator shifts down by a correction which grows upon increasing the linewidth/anharmonicity/damping). However, if this quick estimate cannot be done easily or in short time, this should be left for future work and the paper published as is, so I leave this up to the authors.

- for the 5-th order polynomial in T for specific heat, this can also be derived in Ref. 26 and in Phys. Rev. Research 1, 012010(R) (2019).

Author's Response to Peer Review Comments:

Prof. J.Ll. Tamarit
Department of Physics
Universitat Politècnica de Catalunya
School of Engineering, of Barcelona East (EEBE)
Av. Eduard Maristany, 10-14 08019 Barcelona,
CATALONIA (Spain)
e-mail: josep.lluis.tamarit@upc.edu

Barcelona May, 13th 2022

Dear Editor,

We are submitting a revised version of our manuscript "***The role of optical phonons and anharmonicity in the appearance of the heat capacity boson peak-like anomaly in fully ordered molecular crystals***", for which we have taken into consideration all the comments from the three Referees who have reviewed it.

We appreciate the time devoted by the Referees in reviewing our manuscript, and we are glad for their very positive general evaluation. We list below a point-by-point response to all their comments and suggestions. We also detail, at the end, the nonscientific changes required according to the journal format.

Reviewer: 1

Recommendation: This paper is publishable subject to minor revisions noted. Further review is not needed.

We thank the Referee for his/her careful reviewing work and for acknowledging that this paper is "publishable subject to minor revisions noted".

Comments:
This is a beautiful paper elucidating the origin of the boson peak through a combination of careful measurement in conjunction with cutting edge simulations for a class of model crystalline materials for which the boson peak has been observed in recent measurements. The simulations show that the boson peak in these materials arise from a mode coupling phenomena between the optical and acoustic modes, a coupling that has also been implicated elsewhere in impacting the dispersion relation of the dynamic structure of glass-forming liquids so the effect described is probably of broader significance to the thermodynamics and dynamics of condensed materials. The findings also accord with recent theoretical predictions of Bagglioli and Zaccone (refs. 67 and 67) and suggestions made in some earlier studies. The paper is important because it suggests this mechanism, which was formerly somewhat controversial, in a highly convincing fashion. I strongly endorse the publication of this paper in J. Chem. Phys. Lett.

The only significant criticism that I would make relates to the last sentence of the paper's abstract. which is potentially misleading and which could be simply deleted. There are many physical effects that lead anharmonicity in condensed materials and there is correspondingly no simple unique parameter quantifying the "degree of anharmonicity". The main finding of the paper is that the boson peak can arise purely from anharmonic interactions so structural disorder is not required. It is a separate question of measure or measures of anharmonicity, or properties sensitive to this anharmonicity, determine the height of the boson peak as well as the temperature dependence of the boson peak frequency. The authors should thus delete their overly broad statement at the end of their paper.

Answer: We fully agree with the Reviewer's comment so that we have deleted the last sentence in the Abstract. In passing, by doing this we now fulfil the 150 words limit for the extension of the Abstract.

As an optional matter, I note that the boson peak phenomenon has been noted before in simulations of anharmonic crystalline materials and that such materials are becoming of extreme interest recently in connection of energy conversion, battery and other energy related applications and scientific studies in the contexts of geophysical and astrophysical phenomena. In particular, superionic materials appear to be gaining a lot of recent interest along these lines and I mention a recent molecular dynamics simulation study of the superionic dynamics of crystalline UO2, a major component of reactor fuel, where the boson peak was observed under superionic conditions. Again, there is a broader sphere of problems that the authors results have relevance for that might be mentioned briefly through some referencing. This is left as an option.

Answer: We thank the Reviewer for drawing our attention on such interesting systems where the existence of boson peak anomalies have been evidenced, in particular, the UO_2 crystal. We have added a reference directly related with the Reviewer's comment (Zhang et al., J. Chem. Phys. 150, 174506 (2019), *Superionic UO_2: A model anharmonic crystalline material*), which is relevant to our present work and in general of great scientific and review value (i.e., it contains more than 150 bibliographic references).

Additional Questions:
Urgency: Top 10%

Significance: Top 10%

Novelty: Top 10%

Scholarly Presentation: Top 10%

Is the paper likely to interest a substantial number of physical chemists, not just specialists working in the authors' area of research?: Yes

Reviewer: 2

Recommendation: This paper is publishable subject to minor revisions noted. Further review is not needed.

Comments:
The work presented by Krivchikov and co-workers provides an interesting and fundamentally important study. The study is based on data for crystals, which are used to discuss and elucidate the origin of the so-called "Boson peak", which has for long time been associated with amorphous or partly disordered materials. Based on the results the authors suggest origins of the peak that are unrelated to disorder and corroborate recent theory regarding the Boson peak. The results are important for a deeper understanding of the vibrational density of states in general and the Boson peak in particular. Eventually, this can lead to a more refined theory for heat capacity, and other properties related to the vibrational density of state, beyond that of the Debye theory.

We thank the Referee for his/her careful reviewing work and for acknowledging that this paper is "publishable subject to minor revisions noted". We also thank Referee for his/her positive report comments asserting that our work "*provides an interesting and fundamentally important study*".

Minor issues:
1. Perhaps the authors mean "piling-up" of low-frequency modes instead of "pilling-up".

Answer: We have replaced "pilling-up" by "piling-up" all over the manuscript.

2. I think the comparison of the boson peak between materials with different order is interesting and relevant - hard to understand why it is in the supplementary information. If it is due to length restrictions, then I think it is more interesting than Fig 2, but this assessment is of course up to the authors. In this context, it could also be interesting to include data for amorphous/glassy "BZP", which can be obtained by rapid cooling (supplementary information). In that case, a non-normalized plot would also be interesting.

Answer: We strongly agree with the Reviewer's comment on the interest of our supplementary Fig.S2, thus in the revised manuscript we have moved it to the main text. As for BZP, unfortunately, due to some technical issues, we have not been able to measure the glassy state of this compound (i.e., the involved experimental set-up would require a special cell to confine the liquid state, which is not available to us at this moment). Likewise, we did not find in the literature the experimental C_p values of glassy BZP in the low-temperature range considered in the present study.

3. Supplementary information: There might be a mistake in the description of the source of the materials. It is claimed that some materials was purchased in "USSR", which vanished in 1991.

Answer: The original samples were purchased long time ago from the former USSR.

4. Although the authors find quantitative agreement between the results of the DFT calculations and experimental data, the results seem to imply a rather large Gruneisen parameter. For example, the

(large) increase of the Debye temperature from the experimental value at 1 atm of 82 K to 425 K calculated at 2 GPa indicates a larger Gruneisen parameter than normally found for crystals.

Answer: We thank the Reviewer for making such an interesting comment. Certainly, the large difference between the Debye temperature measured at 1 atm and calculated at 2 GPa suggests a large variation in the phonon frequencies of the analyzed crystals as a function of volume (i.e., the so-called Gruneisen parameter). Nevertheless, it should be noted that the molecular crystals analyzed in this study are highly compressible, essentially due to their notorious organic nature, which means that their volumes vary enormously even under small and moderate pressures. For instance, according to our DFT calculations the volume of the BZP crystal changes by ≈12% when subjected to a pressure of about 2 GPa. Therefore, it may result a bit misleading to draw conclusions on the Gruneisen parameter of a crystal based on the comparison of two Debye temperatures obtained at quite different pressures. On the other hand, the calculation of the full phonon spectrum of the considered materials is computationally so intensive that it is not possible to provide a quantitative estimation of their Gruneisen parameter within a reasonable amount of time. Therefore, we leave a quantitatively rigorous elaboration of this interesting Reviewer's comment to future work.

Additional Questions:
Urgency: High

Significance: High

Novelty: High

Scholarly Presentation: High

Is the paper likely to interest a substantial number of physical chemists, not just specialists working in the authors' area of research?: Yes

"Reviewer #3

I have read this manuscript with much interest. The authors present a compelling experimental and computational study revealing the origin of glasslike anomalies in perfectly ordered anharmonic crystals. The boson peak observed in both VDOS and specific heat is convincingly attributed to either piling up of low-lying optical modes or pseudo-van Hove peaks due to leveling off at avoided crossing between acoustic and low optical modes. This mechanism is going to play an important role for our physico-chemical understanding of glasslike properties in perfectly ordered crystals and for energy materials such as thermoelectrics. I recommend publication of the manuscript with only minor revisions listed below. This is groundbreaking work that certainly belongs to JPCL and will have a great impact on chemistry, physics, and materials science.

We thank the Reviewer for his/her careful reviewing work and for "recommending our work for publication" and considering it as a "groundbreaking work".

- The authors mention that the DFT method is only harmonic. However, from the simulated snapshots of the molecular systems it should be possible to infer also quantitative measures of anharmonicity, see e.g. Phys. Rev. B 105, 184301 (2022) or Phys. Rev. B 105, 014204 (2022). It could be nice, if possible, if the authors could add some measure of anharmonicity in their simulations, especially for the phonons that give rise to the boson peak. Ultimately, the reason why the optical phonons are so low is the anharmonicity (just think of damped harmonic oscillator, when you increase the damping the frequency of the oscillator shifts down by a correction which grows upon increasing the linewidth/anharmonicity/damping). However, if this quick estimate cannot be done easily or in short time, this should be left for future work and the paper published as is, so I leave this up to the authors.

Answer: We thank the Reviewer for his/her very interesting and insightful comment. In the two recent references mentioned by the Reviewer, the authors present [among other things] (1) a heat conductivity study of MoS_2 based on DFT calculations, and (2) a molecular dynamics simulation study of the archetypal metallic glass Cu_xZr_{100-x}. In both works, anharmonicity is quantitatively estimated through different descriptors like the Gruneisen parameter and phonon-phonon interactions (i.e., third-order force constant matrices). However, due to the large number of atoms in the unit cells of the analyzed systems (i.e., ≈100) and their low crystal symmetry, it turns out to be not feasible to perform analogous calculations for the present cases within a reasonable amount of time (i.e., few months). Only the harmonic calculations presented in our manuscript for three different compounds, have required an enormous amount of computational time (i.e., of the order of 2.500.000 CPU hours each system). To provide some numbers, calculation of the full phonon spectrum of BZP have involved about 600 different estimations of all the atomic forces for supercells containing more than 400 atoms, which have demanded a total of about 192 CPU cores running over 24 hours for over 600 repetitions.

With these numbers in mind, the calculation of the phonon-phonon interactions with analogous methods to those employed in work PRB 105, 184301 (2022), for instance, would easily require more than 10.000.000 CPU hours per system (i.e., calculation of anharmonic third-order force constant matrices is computationally much more intensive than calculation of harmonic second-order force constant matrices, typically, by around a factor of 5-10), which is not feasible for us at the moment. Likewise, the work on metallic glass Cu_xZr_{100-x} is based on classical molecular dynamics finite-temperature simulations, which in terms of computational effort and accuracy are not comparable with the techniques employed in the present work (i.e., first-principles zero-temperature DFT methods).

Overall, the calculations proposed by the Reviewer are very interesting but would require titanic computational efforts and some non-trivial code development from our side. Therefore, following the Reviewer's recommendation, we leave those investigations to future work. Nonetheless, in the revised version of our manuscript now we cite the two bibliographic references mentioned by the Reviewer.

- for the 5-th order polynomial in T for specific heat, this can also be derived in Ref. 26 and in Phys. Rev. Research 1, 012010(R) (2019)."

Answer: We thank the Reviewer for drawing our attention on such references, which show a physically insightful way to derive the T^5 dependence of the specific heat that is different from that proposed in Ref. 26. Therefore, we have added the new reference mentioned by the Reviewer in the revised manuscript.

Changes performed according to the editorial requirements

As indicated in the enclosed comments, the reviewers' comments were generally positive, but certain improvements are suggested. I would be pleased to consider further for publication a revised manuscript that addresses the reviewers' concerns. Please also make the **following non-scientific changes:**

1) TOC Graphic: **Provide a TOC image per journal guidelines (2 in x 2 in; on the same page as the abstract) with the heading "TOC Graphic" above the graphic.** The graphic should be in the form of a structure, graph, drawing, photograph, or scheme—or a combination. Non-scientific cartoon-like images or caricatures are discouraged. https://pubsapp.acs.org/paragonplus/submission/toc_abstract_graphics_guidelines.pdf

Answer: TOC image has been included in the first page, before the abstract.

2) Title: In both the main manuscript file and the Supporting Information, **set the title in title case, with the first letter of each principal word capitalized.**

Answer: The format of the title has been modified.

3) **References: In both the main file and the supporting information, fix the style of all references** to use JPCL formatting (check all references carefully). ***JPC Letters reference formatting requires that journal references should contain: () around numbers, author names, article title (titles entirely in title case or entirely in lower case), abbreviated journal title (italicized), year (bolded), volume (italicized), and pages (first-last). Book references should contain author names, book title (in the same pattern), publisher, city, and year.

Answer: Format of the references of the Supporting Information has been modified.

4) Supporting Information: Please number pages in the following format: "S1, S2…"

Answer: Page numbers have been introduced in the Supporting Information.

5) **Cover Art: You have uploaded a cover art file. Please upload a cover art caption file, and be sure to indicate "Yes" to the custom question regarding cover art.**

Answer: The option of a Cover Art has been withdrawn.

In addition, all the format requirements have been taken into account.
